# Green Synthesis, Characterization, Antioxidant, Antibacterial and Enzyme Inhibition Effects of Chestnut (*Castanea sativa*) Honey-Mediated Silver Nanoparticles

**DOI:** 10.3390/molecules28062762

**Published:** 2023-03-18

**Authors:** Merve Keskin, Gülşen Kaya, Sinan Bayram, Anna Kurek-Górecka, Paweł Olczyk

**Affiliations:** 1 Vocational School of Health Services, Bilecik Seyh Edebali University, Bilecik 11100, Türkiye; 2 Scientific and Technological Research Center, Inonu University, Malatya 44000, Türkiye; 3 Vocational School of Health Services, Bayburt University, Bayburt 69000, Türkiye; 4 Department of Community Pharmacy, Faculty of Pharmaceutical Sciences in Sosnowiec, Medical University of Silesia in Katowice, 40-055 Katowice, Poland

**Keywords:** green synthesis, honey, silver, antioxidant, antibacterial, enzyme inhibition

## Abstract

In this study, chestnut honey-based silver nanoparticles (CH-AgNPs) were synthesized at different temperatures (30, 60 and 90 °C) and these nanoparticles were characterized by different techniques such as UV–vis spectrophotometer, Fourier transform infrared spectroscopy (FTIR), scanning electron microscopy (SEM) and energy dispersive X-ray (EDX). The DPPH free radical scavenging assay was used to determine the antioxidant activity of the obtained nanoparticles. The inhibition effects of these nanoparticles for some clinically important enzymes such as myeloperoxidase and collagenase were investigated. In addition, the disk diffusion method (DDM), agar well diffusion (AWD), and minimum inhibitory concentration (MIC) and minimum bactericidal concentration (MBC) techniques were used to determine the antibacterial activity of CH-AgNPs. In honey-based silver nanoparticle production processes using green synthesis, it was determined that the nanoparticle sizes decreased from 55 to 27 nm with an increase in temperature. In addition, it was determined that the rate of inhibition of myeloperoxidase (36.4% to 34.0%) and collagenase enzymes (74.2% to 68.7%) increased with a decrease in particle size. As a result of the antibacterial activity tests, it was observed that CH-AgNPs have antibacterial activity against all target pathogens including Gram-positive and Gram-negative bacteria. The obtained results show that CH-AgNPs produced using chestnut honey have the potential to be used in fields such as medicine, pharmacy and cosmetic technology.

## 1. Introduction

Nanotechnology is a multidisciplinary science that aims to design and synthesize nanoparticles (NPs) to obtain functional materials. Nanoparticles are extremely small sized particles in the range 1–100 nm. Their unique by chemical, biological and physical properties lead to their wide range of applications in electronics, sensors, analysis, energy, cosmetics, food and pharmaceutical industries [1,2,3]. They are particularly important in finding new pathways for the synthesis of medicines [1].

Biocompatible metallic nanoparticles (gold, silver, titanium, iron, etc.) are used as biomaterials in products such as shampoo, soap, hand cream, toothpaste, textile, and cosmetics due to their mechanical properties, thermal conductivity, and electrical and biological properties [4]. They are also used in the pharmaceutical industry, catalysis, cancer diagnosis, and as antimicrobial agents [5].

NPs are synthesized by biological, physical and chemical methods. In the synthesis of silver nanoparticles using chemical methods, stabilizing agents, thermal decomposition organic solvents, chemical reaction and photoreduction, in reverse micelles and radiation are very harmful for the environment. There are many disadvantages of physical and chemical synthesis such as long production times, toxic products, poor particle stability, expense and environmental effects. Green technology (green synthesis) was developed as an alternative synthesis technique for chemical and physical techniques due to its advantages. Green technology (the biological method) is preferred to other (physical and chemical) methods because it is environmentally friendly and cost-effective [6,7]. In green synthesis, toxic solvents are avoided. Therefore, the process is biocompatible and has higher stability. Biological sources that can be used in green synthesis include bacteria [8], seaweeds [9], fungi [10] and plants [11]. In the synthesis of silver nanoparticles, the use of plant sources is more economical and the application processes are simpler, attracting more attention recently [12,13,14]. AgNPs obtained by green synthesis are also important for living applications due to their biocompatibility [15]. Since natural products often have antimicrobial activity, the nanoparticles have also antimicrobial, antioxidant and enzyme inhibition properties through synergistic or additive effects.

Honey is an important natural bee product used as a food and medicine. Honey is frequently used—but not only—as a natural medicine in different apitherapeutic applications such as wound healing, cancer, and acute coronary syndrome [16]. It is collected by honey bees (*Apis mellifera* L.) from nectar found in the flowers of plants or the sweet secretions produced by insects living on some plants, and stored in the honeycomb cells. Different colors, tastes and compositions can be observed in honey obtained from different botanical sources. Honey is an important source of natural antioxidants such as organic acids, amino acids, proteins, phenolic compounds and flavonoids. In general, honey consist of 80% carbohydrates (35% glucose, 40% fructose, and 5% sucrose) and 20% water with some other active biocompounds (e.g., amino acids, vitamins, minerals, enzymes, organic acids, flavonoids, and phenolic compounds) [17]. The synthesis of AgNPs could be triggered with the use of aqueous honey solutions, where the sugars contained—mainly mono- and disaccharides—play a crucial role as reducing agents. The antioxidant activity of honey depends on its bioactive compounds. Based on the literature, it was reported that dark-colored honeys have higher phenolic content and thus have higher antioxidant activity [18]. Chestnut honey is a dark-colored honey with higher antioxidant capacity than many honey types such as lavender, linden, sunflower and multifloral blossom honey. In the literature, it was reported that colloids, which are responsible for the bioactivity of honey, constituted from 0.1% to 1% of honey weight and proteins constituted 54% [19]. These colloidal molecules of dark honey (as chestnut honey) provide colloidal stability [19] even if dilution with water is doubled. The protein–carbohydrate interactions and the occurrence of polyphenol–protein complexes are important for aggregated structures of AgNPs. In recent years, studies focused on the prevention of aging, degenerative heart and nervous system diseases and the protection of foods from oxidation, especially related to human health, have increased by utilizing the antioxidant properties of chestnut honey [16,20]. In addition, the use of honey in dentistry is becoming widespread. It was determined that honey inhibits oral pathogens and thus reduces the formation of dental plaque, and it was reported that it controls biofilm accumulations in the teeth [21]. In the literature, there are many studies that involve the synthesis of honey-based silver nanoparticles but none of them involve chestnut honey [14,22,23,24,25,26,27]. It was thought that chestnut honey would be a good precursor compared to other honey types, with its high antioxidant activity.

In this study, chestnut honey was used as a precursor for the synthesis of honey based on silver nanoparticles. The effect of temperature on synthesis was examined. The chestnut honey based on silver nanoparticles (CH-AgNPs) synthesized at different temperatures were characterized and their inhibition properties of clinically important myeloperoxidase and collagenase enzymes and antibacterial activities on different microorganisms were determined.

## 2. Results

**Figure 1:**
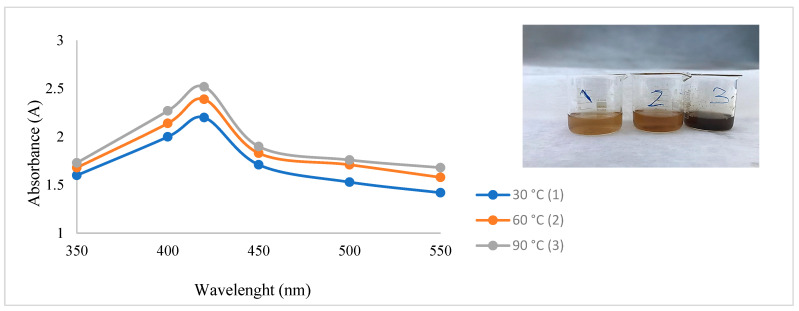
UV spectrum of CH-AgNPs after 30 min.

**Figure 2:**
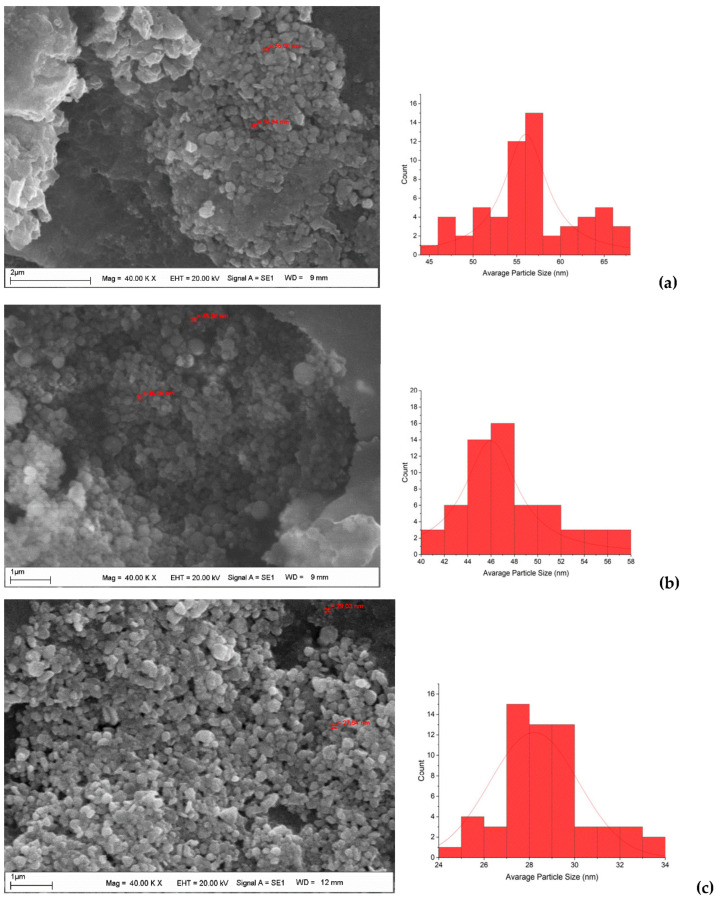
SEM images and histograms of CH-AgNPs at 30 (**a**), 60 (**b**) and 90 °C (**c**).

**Figure 3:**
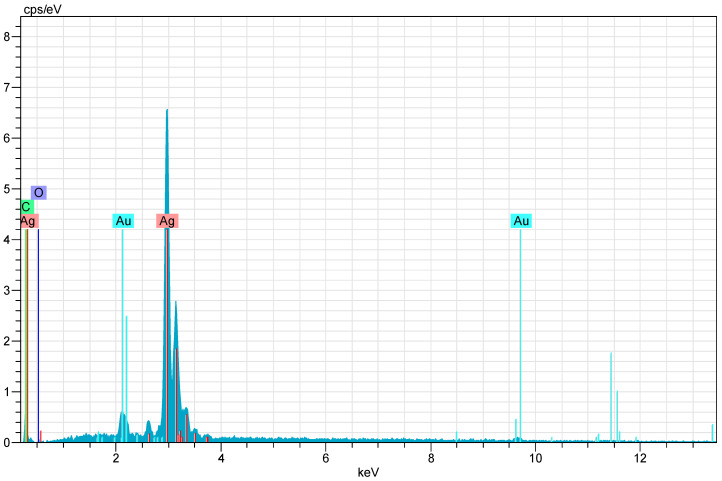
X-ray (EDX) images of CH-AgNPs.

After CH-AgNPs were synthesized (Figure 1), they were characterized using various techniques in terms of shape, size, distribution, homogeneity and surface morphology. CH-AgNPs were characterized by UV–vis absorption spectroscopy (Figure 1) and it was determined that silver nanoparticles obtained at three different temperatures showed absorbance at 420, 425 and 427 nm. It was determined that the rate of change in color to dark brown was directly proportional to the increase in temperature. This situation was found to be compatible with the literature [28,29]. Fourier transmission infrared (FTIR) spectroscopy, energy dispersive X-ray analysis (EDX) and scanning electron microscopy (SEM) were also used for characterization of CH-AgNPs. CH-AgNPs synthesized at three different temperatures (30, 60, 90 °C) by green synthesis and particle sizes were found below 60 nm (Figure 2) in the scanning electron microscope at all three different temperatures and had a 2.8 keV peak, which showed the presence of Ag (Figure 3). The variation of reaction temperatures allows the particle size to decrease from 55 to 27 nm. The smallest particle size (27–29 nm) was observed at the highest reaction temperature (Figure 2). EDX is applied to analyze the dispersed size distribution in the liquid and the principal components of NPs, respectively [30,31]. However, when the energy dispersive X-ray (EDX) analysis was examined, it was determined that the nanoparticles were mainly composed of elemental silver (normalized atomic value 62.67%), and the C and O contents were relatively lower (Table 1).

**Figure 4:**
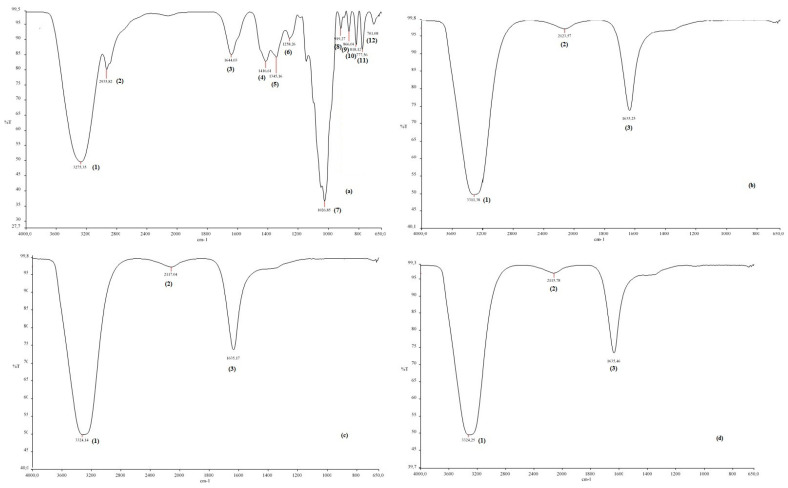
FTIR spectra of chestnut honey (**a**) and CH-AgNPs at 30 (**b**), 60 (**c**) and 90 °C) (**d**).

To determine the functional groups of honey and CH-AgNPs, FTIR spectroscopy was used [32]. Proteins in honey act as a capping agent, stabilizing the nanoparticles, while fructose acts as a reducing agent [33]. In this study, the Fourier transform infrared spectroscopy (FTIR) spectrum of the control sample and the spectra of CH-AgNPs synthesized at three different temperatures (30, 60, 90 °C) were compared (Figure 4, Table 2). The wide peak at approximately 3275–3300 cm^−1^ in the spectrum indicates the presence of an O-H group. In addition, peaks at 2935, 1644, 1416, 1345, 1258, and 1026 cm^−1^ were found in the spectrum of the control sample. These peaks are associated with stretching vibrations of alkane, ketone, alkene, nitro compounds corresponding to C=C, C=O, O-H, C-H, C-N, and -COOH groups in the control sample. The disappearance of the bands at 2935 and 1026 cm^−1^ in the control sample after nanoparticle synthesis supports possible interactions between molecules and that these functional groups that are depleted in the stabilization of nanoparticles. The fact that the peak at 1026 cm^−1^ in the control sample is not observed in the FTIR spectra of AgNPs synthesized using three different temperatures proves that the proteins bind to silver nanoparticles via the carboxylate group [34]. Some studies support that molecules bind to nanoparticles through functional groups, thereby stabilizing them by wrapping around them [14].

In this study, the effect of temperature on the formation of nanoparticles and the biological activities of the obtained nanoparticles were determined. Our results indicated that CH-AgNPs had better antioxidant activity than chestnut honey (Table 3). It was determined that nanoparticles obtained at a high temperature inhibit collagenase and myeloperoxidase enzymes better because of the surface area of nanoparticles.

In a study, it was stated that the obtained nanoparticles showed 83.5% DPPH activity and inhibited the collagenase enzyme by 23.68% [34]. When the literature is examined, it is seen that silver nanoparticles obtained by using different sources have antioxidant and anti-inflammatory properties (Table 4).

**Figure 5:**
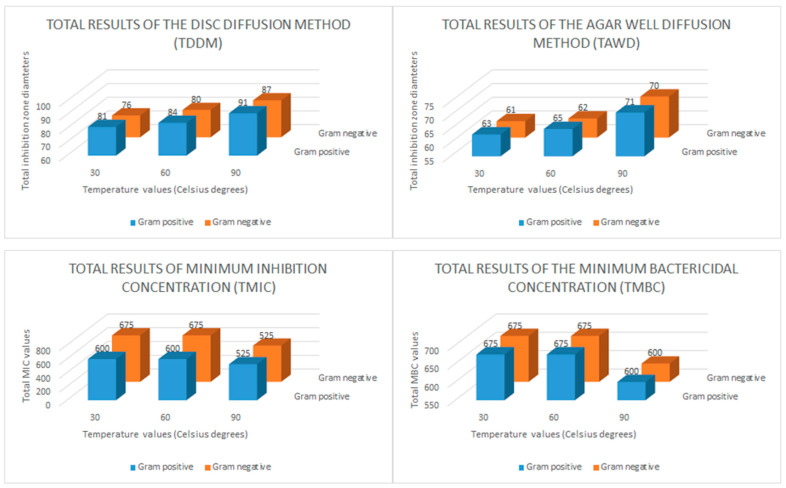
Bar graph of the in vitro antibacterial activity of the chestnut honey-based silver nanoparticles (CH-AgNPs). Total results of the disc diffusion method (TDDM: mm), total results of the agar well diffusion method (TAWD: mm), total results of the minimum inhibition concentrations (TMIC: μg/mL) and total results of the minimum bactericidal concentration (TMBC: μg/mL).

The disk diffusion method (DDM), the agar well diffusion (AWD) method, and minimum inhibition concentration (MIC) and minimum bactericidal concentration (MBC) tests were applied to determine the antibacterial effects of the CH-AgNP samples. During the disk diffusion method, 10 μL CH-AgNP sample was impregnated into discs (300 μg/mL). The results obtained after this assay showed that CH-AgNP samples inhibited all Gram (+) and Gram (−) pathogen samples, and it was observed that the diameters of these inhibition zones varied between 14 and 20 mm (Table 5). After that, in the AWD assay process, 8 mm diameter wells were drilled into the media (MHA, Merck, Darmstadt, Germany) and a previously prepared CH-AgNPs suspension (50 μL) at a concentration of 300 μg/mL was transferred to these wells. The obtained results after this application showed that CH-AgNP samples inhibit all target pathogen samples and it was observed that these inhibition zone diameters varied between 11 and 16 mm (Table 5). After AWD assays, MIC and MBC values were determined by the micro broth dilution method and it was observed that these values varied between 75 and 150 μg/mL. In order to easily interpret the data obtained from antibacterial activity tests, total results of the disk diffusion method (TDDM), total results of the agar well diffusion method (TAWD), total results of the minimum inhibition concentrations (TMIC) and total results of the minimum bactericidal concentration (TMBC) values were calculated. These total results are given in Figure 5.

## 3. Discussion

Phenolic compounds, vitamins and sugars (fructose and glucose) that have been found in honey are important reducing agents involved in green synthesis. These biomolecules found in the honey provide the reduction of Ag^+^ ions to Ag^0^ in only one step. These molecules of dark honey (as chestnut honey) also provide colloidal stability [43] even if dilution with water is doubled. The protein–carbohydrate interactions and the occurrence of polyphenol–protein complexes are important for aggregated structures of AgNPs. During the synthesis, there could be non-covalent and covalent protein–protein and protein–polyphenol interactions, van der Waals bonds, hydrogen bonds and at low temperature. These types of interactions leading to the formation of aggregates can significantly reduce the availability of reducing components in the honey solution. In addition, the increased temperature broke van der Waals and hydrogen bonds, thereby initiating the processes of monomerization and causing significant acceleration of Ag^+^ ion reduction.

**Figure 6:**
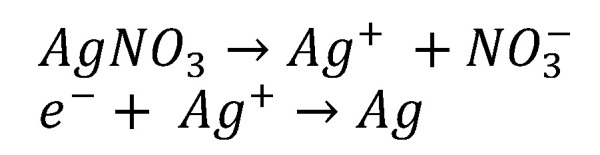
Mechanism of reducing Ag^+^ ion to metallic Ag [45].

The bioreduction of Ag^+^ ions to AgNPs occurs due to the presence of phenolic compounds and reducing sugars. In addition, other compounds such as flavonoids and proanthocyanidins may also cause the reduction of Ag^+^ ions to AgNPs and play a role as a capping agent to prevent agglomeration. The hydroxyl groups of flavonoids have a greater ability to bind with silver ions and act as reducing agents [44]. Different types of antioxidants and biomolecules present in chestnut honey act as donors and synergistically reduce Ag metal ions (Figure 6).

In a study performed by Al-Brahim and Mohammed [46], they stated that honey samples obtained from different regions showed absorbance at 441 and 446 nm, and the average particle sizes were 50.5 and 98.2 nm. In another study, it was reported that silver nanoparticles obtained from Egyptian honey showed absorbance at between 425 and 450 nm [47]. Matar et al. [14] reported that the silver nanoparticles obtained using Turkish honey showed absorbance at 443 and 456 nm, and the average particle size of nanoparticles was 14.3 and 14.7 nm [14]. Czernel et al. [19] reported that the nanoparticles obtained in the above study showed absorbance at 414, 413, and 407 nm, and the particle sizes were measured with an average particle size of 42–55, 55–75, 55–70, and 66–80 nm. It was clear that honey-based silver nanoparticles had different UV absorbance and particle sizes [14,19,47].

In a study, the variation in particle size with temperature of silver nanoparticles was investigated [48]. Fayaz et al. synthesized the nanoparticles at three different temperatures (10, 27, and 40 °C). They observed that an increase in reaction temperature resulted in a decrease in particle size, while a decrease in reaction temperature resulted in an increase in particle size. The optical absorption peak is observed at approximately 3 eV, which is typical for metallic silver nano crystalline absorption due to surface plasmon resonance. This result is compatible with our study. The lower particle size formation at a higher temperature is due to the increased reaction rate at a higher temperature. As the reaction rate increases, the reactants are consumed faster, hence reactant depletion occurs, leading to the formation of smaller nanoparticles and a narrow size distribution at a higher temperature [49].

Ghramh et al. [27] reported that the peak spectra at approximately 3289 cm^−1^ present an O-H group [27]. The weak bands at 2967, 1733, 1522, 1033, 722 and 600 cm^−1^ were related to the stretching vibration of the alkane, ketone, and alkene corresponding C-H, C=O, C=C, N-O, C-O and C-Cl. After synthesis, the bands shifted to the lower-intensity bands of 3200, 2967, 1758, 1000, 711, and 600 cm^−1^, which could be assumed to be used in the reduction and capping of silver nanoparticles. The band at 1333 cm^−1^ was C-N stretching of the aromatic amine as a by-product of the reduction process. The disappearance of the band at 1522 cm^−1^ means that the alkene was depleted in the reduction and stabilization of the AgNPs [27]. The peaks obtained at 2922.8 and 2922.7 cm^−1^ represented stretching in the C-H bonds of aromatic compounds [50]. Peaks ranging from 1742.6 to 1653.1 cm^−1^ represent C=O linkages in aldehydes, while peaks at 1456.1 cm^−1^ indicate C-H stretching in alkenes [51,52]. The peaks could be attributed to N–O asymmetric stretching vibrations [51,52]. The peak at 1387.3 cm^−1^ indicated -C-O-like phenol groups [53], while the peaks at 1045.0 and 1036.7 cm^−1^ represent C-O [54]. Our results were similar to data from other studies. Haiza et al. [25] reported that the peak spectra at approximately 1647 and 1576 cm^−1^ present amide I and II bands of protein. The weak bond at 1079 cm^−1^ arises from the C-O-C symmetric bending and C-O-H bending vibrations of protein in the honey [25]. In another study, it was reported that the peak at approximately 3366 cm^−1^ related to the functional groups of phenols’ OH and the peak in the range 2892–2938 cm^−1^ belongs to the CH groups of aldehydes [22].

The inhibition of myeloperoxidase and collagenase enzymes, which have a role in skin regeneration, wound healing, cardiovascular diseases, inflammatory diseases, neurodegenerative diseases, kidney diseases and immune-mediated diseases, is very important for developing and discovering new drugs [55,56]. Due to the side effects of synthetic medicines, it is very important to develop natural products such as plant-based drugs, as well as different formulations of cosmetics such as creams and sprays. Nanoparticles obtained using silver, known for its antimicrobial properties, are widely used in food additives, pharmaceuticals, personal care products and cosmetics.

When we compared the inhibition effects of CH-AgNPs with the standard inhibitors, it was clear that CH-AgNPs had a better inhibition effect than standard inhibitors. In a study, blackcurrant-based silver nanoparticles were synthesized [57]. It was stated that the total phenolic content of the obtained blackcurrant extract was 74.96 mg GAE/m^3^, and the DPPH activity was 84.17%. Results of mentioned study demonstrate that the obtained silver nanoparticles reduced myeloperoxidase enzyme activity [57]. In another study, *Eucalyptus camaldulensis*-based silver nanoparticles were obtained, and the inhibition effects of the obtained nanoparticles on the collagenase enzyme were also determined. The total amount of phenolic substance was 434.8 mg/g GA [58]. They stated that the obtained nanoparticles inhibited the collagenase enzyme by 75%on average; in addition, a correlation between the inhibition of the collagenase enzyme and the amount of total phenolic substance was observed [58]. In another study, *Aquilegia pubiflora*- based silver nanoparticles were synthesized [59]. It was reported that Ag-NPs had excellent inhibitory potential on enzymes [59].

The antimicrobial effect of silver ions and silver salts has been known for many years. Using the biocompatible and biodegradable materials for medical and pharmacological purposes provide significant advantages. Reducing the particle size is an ideal technique in order to increase the biocompatible properties of silver salts and ions and these techniques have been widely used in recent years. For this purpose, in many different studies, bioactive properties of nanoscale silver particles have been investigated. Today, the demand for nanoparticle production by using green synthesis techniques continues to increase day by day and these green synthesis techniques do not pose a threat for human health and environmental health. In addition, use of Ag nanoparticles prepared by these green synthesis methods in antimicrobial applications can also offer an alternative solution for the increasing problem of antibiotic resistance. The antimicrobial properties of silver salts and silver NPs are based on more than one mechanism. These nanoparticles increase the permeability of the cell membrane; and as a result of this process, they cause the production of reactive oxygen species such as peroxides, superoxides and hydroxyl radicals. In addition, Ag ions can also interrupt the replication of nucleic acids [60,61,62,63].

When we evaluate in terms of the total results of the disk diffusion method (DDM), it is seen that the CH-AgNP sample prepared at 30 °C creates a total inhibition zone of 81 mm for five different Gram-positive bacteria and 76 mm for five different Gram-negative bacteria. It is seen that these values are measured as 91–87 mm, respectively, for the CH-AgNP sample prepared at 90 °C. These results show that CH-AgNP samples prepared at 90 °C have a much higher antibacterial effect compared to CH-AgNP samples prepared at 30 °C. In addition, when we evaluated the total results of the agar well diffusion method (TAWD), it is seen that the CH-AgNP sample prepared at 30 °C creates a total inhibition zone of 63 mm for five different Gram-positive bacteria and 61 mm for five different Gram-negative bacteria. It is seen that these values are measured as 71–70 mm, respectively, for the CH-AgNP sample prepared at 90 °C. These results reveal that the CH-AgNP sample prepared at 90 °C has more effective antibacterial activity compared to the sample prepared at 30 °C. In addition, it is seen that the TMIC value of the CH-AgNP sample prepared at 30 °C was 600 μg/mL for five different Gram-positive bacteria and 675 μg/mL for five different Gram-negative bacteria. For the CH-AgNP sample prepared at 90 °C, these values were found to be 525–450 μg/mL, respectively. When the TMIC values are compared, it is observed that the CH-AgNP sample prepared at 90 °C has a lower TMIC value compared to the CH-AgNP sample prepared at 30 °C. These results confirm again that the CH-AgNP sample prepared at 90 °C has a greater antibacterial effect than the CH-AgNP sample prepared at 30 °C. Finally, it was observed that the CH-AgNP sample prepared at 90 °C had TMBC values of 600 μg/mL for both Gram-positive and Gram-negative bacteria, while these values were 675 μg/mL for the CH-AgNP sample prepared at 30 °C. All these results confirm that the CH-AgNP sample prepared at 90 °C has a smaller particle size and greater antibacterial effect compared to the CH-AgNP sample prepared at 30 °C.

In a study conducted by Annadhasan et al. [64], the sunlight-induced rapid synthesis of AgNPs was carried out using sodium salt of N-cholyl amino acids and the antimicrobial activity of these AgNPs was determined [64]. The obtained results in the above study showed that the produced AgNPs have significant antimicrobial activity against target pathogens (*Escherichia coli, Staphylococcus aureus* and *Pseudomonas aeruginosa*) and it was reported that the obtained MIC values ranged from 1.75 to 3.5 μg/mL. In the described study, the anticandidal activity of AgNPs against three different *Candida* species (*Candida albicans, Candida krusei* and *Candida tropicalis*) was also determined, and the strong antifungal activity of AgNPs against *Candida* species was determined [64]. 

In another study, the antimicrobial activity of AgNPs produced using sunlight and cationic surfactants was determined [65]. AgNPs showed strong antimicrobial activity against different microorganism groups (*Pseudomonas aeruginosa, Candida krusei, Sarcina lutea, Bacillus pumilus, Micrococcus luteus, Candida albicans,* and *Penicillium chrysogenum*).

In addition to these studies, procyanidin-capped AgNPs were biosynthesized, characterized, and their biological activities were evaluated [66]. Researchers used procyanidin dimers and *Leucosidea sericea* total extract for this purpose. In this study, *Pseudomonas aeruginosa, Staphylococcus aureus, Bacillus cereus, Salmonella enterica, Escherichia coli* and *Serratia marcescens* were selected as target pathogens and it was determined that the obtained MIC values ranged from 15.63 to 125 μg/mL [66]. When evaluated in terms of antibacterial activity, it was observed that these AgNP samples produced with an environmentally-friendly method give an effective result against all Gram (+) and Gram (−) bacteria and these results are consistent with the literature data [34,67,68,69,70,71]. 

## 4. Materials and Methods

### 4.1. Green Synthesis of Chestnut (Castanea Sativa) Honey-Based Silver Nanoparticles (CH-AgNPs)

CH-AgNPs were synthesized according to Baran et al. [72], with minor modifications [72]. For this purpose, chestnut honey was purchased from a local beekeeper from Kastamonu, Turkey in 2020. The honey sample was dissolved in deionized water at a 1:1 ratio at room temperature. The extract was mixed with 5 mM silver nitrate (AgNO_3_) solution in a dark flask (1:1). The mixture was stirred for ~2 h at room temperature. The change in color to dark brown was noted. The formation of silver nanoparticles was affirmed by scanning of UV absorption between 250 and 750 nm. Honey-based silver nanoparticles were synthesized using the same method at 30, 60 and 90 °C separately. Once CH-AgNPs synthesis was completed, the solution was centrifuged at 9000 rpm for 10 min at room temperature. The purpose of this process was to separate the synthesized nanoparticles from honey residues. The solid fraction obtained at the end of centrifugation was washed several times with distilled water and the resulting residue (CH-AgNPs) was dried in an oven at 75 °C for 12 h [72,73].

### 4.2. Characterization of CH-AgNPs

In order to determine the optical properties of the particles obtained, absorbance measurements were made with a UV spectrophotometer and the highest absorbance value of the particles was recorded. The functional groups of bioactive components were evaluated with Fourier transform infrared spectroscopy (FTIR). The nanoparticles formed were characterized by using scanning electron microscopy (SEM) to determine the particle size and shape; in addition, energy dispersive X-ray (EDX) was used to determine the metallic chemical composition [72,74]. A Au/Pd coating was applied to the samples in order to increase conductivity for SEM/EDX analysis.

### 4.3. The Total Phenolic Content of Chestnut (Castanea Sativa) Honey Extract and CH-AgNPs

The total phenolic content of natural products could be determined according to the Folin method [69,70]. The phenolic compounds and Folin–Ciocâlteu reagent become a colored complex and give a maximum absorbance at 765 nm. Gallic acid is usually used as a standard to produce a calibration curve [75,76]. The results are represented in milligrams of GA equivalent per gram (mg GAE/g). The analyses were performed in triplicate.

### 4.4. DPPH·Free Radical Scavenging Activity

The DPPH· free radical scavenging assay was performed using the method described by Cuendet et al. [77]. CH-AgNPs solutions were prepared at different concentrations (50–150 µg/mL). The DPPH· solutions prepared in absolute methanol and substrates were mixed and incubated separately at room temperature. A decrease in the absorbance at 517 nm was recorded after incubation. The IC_50_ results are expressed in milligrams per milliliter (mg/mL) (y = 0.50 × 10^−0.00x^, R^2^: 0.982). The analyses were performed in triplicate. Trolox was used as a standard.

### 4.5. Collagenase Inhibition

The inhibition of collagenase activity was determined according to Tu et al. [78]. FALGPA (N-(3-[2-Furyl]acryloyl)-Leu-Gly-Pro-Ala) was used as a collagenase substrate. The collagenase enzyme was incubated for 15 min at room temperature, using different amounts of honey extract and CH-AgNPs, in 50 mM Tris-HCl (pH 7.5) buffer containing 10 mM CaCl_2_ and 400 mM NaCl. Then, a 0.8 mM solution of specific collagenase substrate FALGPA was added to the mixture and incubated at 37 °C for 20 min. The total volume of the reaction mixture was 150 µL. A blank tube was prepared by adding distilled water instead of the enzyme. At the end of the relevant time, the absorbance of the tubes was recorded at 340 nm [78]. The analyses were performed in triplicate. The inhibition percentage of the CH-AgNPs was calculated according to the formula below. Oleanolic acid was used as a standard inhibitor.

Collagenase Inhibition (%) = (A−B)/A × 100 (1)

A is the enzyme activity without the inhibitor, and B is the enzyme activity in the presence of the inhibitor.

### 4.6. Myeloperoxidase (MPO) Inhibition

The inhibitory effect of honey extract and CH-AgNPs on MPO activity was determined according to Khalil et al. [79]. Guaiacol was used as the substrate. The MPO (2.5 nM) enzyme was pre-incubated with 1 mL of 50 mM phosphate buffer (pH 7.4) containing 0.5 mM H_2_O_2_ and honey extract and CH-AgNPs at different concentrations for 15 min. Then, by adding 1 mM guaiacol solution to the reaction tubes, the reaction was started at 37 °C and the absorbance was recorded at 470 nm for 3 min. [79]. The analyses were performed in triplicate. The inhibition percentage of the CH-AgNPs was calculated according to Formula (1). Quercetin was used as a standard inhibitor.

### 4.7. Antibacterial Activity

#### 4.7.1. Microorganisms and Growth Conditions

Ten different pathogenic bacterial strains (five Gram (+) and five Gram (−)) were used to determine the in vitro antibacterial effects of CH-AgNPs. Initially, the selected pathogenic bacteria strains were cultured in Nutrient Broth medium (NB, Oxoid) for 24 h at 37 °C. After this incubation time, the prepared bacterial suspensions were adjusted to a 0.5 McFarland standard turbidity (10^6^ CFU/mL) and used as inoculum for the determination of the antibacterial activity of CH-AgNP samples [80]. The pathogenic bacterial strains used in this study were obtained from the Bayburt University Vocational School of Health Services, Department of Medical Services and Techniques.

#### 4.7.2. The Disk Diffusion Method (DDM)

Initially, Mueller–Hinton Agar (MHA) medium was sterilized in an autoclave and allowed to cool to 50 °C degrees at room temperature. After this process, 25 mL of MHA medium was transferred to each sterile Petri dish and allowed to cool for 2 h at room temperature. After solidification of the media in Petri dishes, using a sterile swab, the bacterial suspension (10^6^ CFU/mL) was inoculated to cover the entire surface of the medium. After that, blank cellulose discs (6 mm diameter: OXOID) were carefully placed on the surface of the medium at ideal intervals. Immediately after this process, 10 µL of CH-AgNP samples were transferred onto blank discs. At the end of these procedures, Petri dishes were incubated at 37 °C for 24 h. The zones observed around the discs were then measured with a Vernier caliper and recorded [81].

#### 4.7.3. The Agar Well Diffusion Method (AWD)

MHA media were prepared as described above in the disk diffusion method. After solidification of the media in Petri dishes, using a sterile cork borer, 8 mm diameter wells were cut into MHA agar and media were inoculated with a sterile cotton swab [82,83]. After these inoculation processes, 50 µL of the CH-AgNP samples was transferred to the wells and incubated at 37 °C for 24 h. At the end of all these processes, the inhibition zones around the wells were measured with a Vernier caliper and recorded [49,84]. Each assay was performed in duplicate.

#### 4.7.4. The Minimum Inhibitory Concentration (MIC)

In order to determine the minimum inhibitory concentration of the CH-AgNP samples, the micro broth dilution method was used [82]. A volume of 95 μL of sterile MHB medium and 5 μL of the inoculum were added to all wells and completed to 100 μL. Then, 100 μL of the previously prepared CH-AgNP samples was added and mixed thoroughly using a multichannel pipette. Following these processes, 100 μL of the suspension samples was taken from the first wells and transferred to the second wells by a multichannel pipette. This transfer process was repeated sequentially to the 8th well and the concentrations of the CH-AgNP samples in each well were serially diluted. After completing this series of micro dilution, the prepared 96-well polystyrene micro titer plates were recorded at a wavelength of 600 nm using a plate reader (Thermo, Multiskan Go, Waltham, MA, USA) and incubated at 37 °C for 24 h at 60 rpm with an orbital shaker (Heidolph Instruments, Schwabach, Germany). The micro plates were again measured at 600 nm and the obtained results were recorded. Lastly, the lowest concentrations of CH-AgNP samples that inhibit target pathogenic bacteria were determined as the MIC [82].

#### 4.7.5. The Minimum Bactericidal Concentration (MBC)

The minimum bactericidal concentration (MBC) values were determined according to Ecem-Bayram et al. with minor modifications [85]. After the determination of the MIC values, 7 μL suspension samples were taken from each well of the micro plate using a micropipette and transferred to the MHA media in sequence. Following these transfer processes, the Petri plates were incubated at 37 °C for 24 h. After this incubation time, the lowest concentration that did not grow a bacterial colony was accepted as the MBC [85].

## 5. Conclusions

Nowadays, the use of silver nanoparticles in numerous industrial areas is becoming more common. In this study, environmentally-friendly, economical, fast, and non-toxic honey-based silver nanoparticles were synthesized at different temperatures (30, 60, and 90 °C). The antioxidant, antibacterial, and enzyme inhibition properties of these nanoparticles formed by green synthesis were determined. Chestnut honey was used due to its rich content of phenolics, flavonoids, vitamins, and sugar components. The obtained results showed that the prepared silver nanoparticles (CH-AgNPs) have clinical importance in terms of the inhibition properties of myeloperoxidase and collagenase enzymes in addition to antibacterial and antioxidant effects. In conclusion, the obtained results show that CH-AgNPs have the potential to be used in different fields such as apitherapy, cosmetic technology, medicine, and pharmacy.

## Figures and Tables

**Table 1:** Normalized element composition of CH-AgNPs.

El	AN	Series	unn.	C Norm.	C Atom.	C Error
			[wt.-%]	[wt.-%]	[at.-%]	[%]
Ag	47	L-series	81.90	93.46	62.67	2.6
C	6	K-series	4.54	5.18	31.21	1.2
O	8	K-series	1.19	1.35	6.12	1.0
Total:			87.62	100.00	100.00	

**Table 2:** The list of FTIR peaks.

Peak Number	C. Honey Extract (cm^−1^)	30 °C (cm^−1^)	60 °C (cm^−1^)	90 °C (cm^−1^)
**1**	3275.35	3311.38	3324.14	3324.24
**2**	2935.82	2123.57	2117.04	2115.78
**3**	1644.03	1635.25	1635.17	1635.46
**4**	1416.61	-	-	-
**5**	1345.16	-	-	-
**6**	1258.26	-	-	-
**7**	1026.85	-	-	-
**8**	919.27	-	-	-
**9**	866.04	-	-	-
**10**	818.12	-	-	-
**11**	777.56	-	-	-
**12**	701.08	-	-	-

**Table 3:** Biochemical and enzyme inhibition properties of honey extract and CH-AgNPs.

	Total Phenolic Content (mg GAE/g)	DPPH·IC_50_ Value (mg/mL)	Collagenase Inhibition (%)	Myloperoxidase Inhibition (%)
**Honey extract**	1.21 ± 0.02 ^a^	21.37 ± 0.01 ^a^	60.0 ± 1.4 ^a^	30.0 ± 1.0 ^a^
**CH-AgNPs (30 °C)**	0.92 ± 0.01 ^b^	15.08 ± 0.02 ^b^	68.7 ± 1.1 ^b^	34.0 ± 1.1 ^b^
**CH-AgNPs (60 °C)**	0.87 ± 0.01 ^c^	14.67 ± 0.01 ^c^	71.4 ± 1.3 ^c^	35.2 ± 1.0 ^c^
**CH-AgNPs (90 °C)**	0.84 ± 0.01 ^d^	13.71 ± 0.02 ^d^	74.2 ± 1.1 ^d^	36.4 ± 1.2 ^d^
**Trolox**	-	0.009 ± 0.001	-	-
**Oleanolic acid**	-	-	28.4 ± 1.98	-
**Quercetin**	-	-	-	48.6 ± 1.1

Means of each group with the different letter(s) are significantly different (*p* < 0.05).

**Table 4:** Synthesis of AgNPs by using different sources.

Plant or Biological Source	Reaction Conditions	Average Size (nm)	Shape	Biomedical Application	Ref.
Nigella sativa (black cumin, seeds)	75 °C; 2 days	3.47	Spherical	Anticancer activity against human breast (MDA-MB-231) and cervical (HeLa) cancer cells Bactericidal against Gram-negative and Gram-positive bacteria	[35]
Green tea (powder extract)	50 °C; 4 h	2	Spherical	Anticancer activity against two human colon cancer cell lines (SW480 and SW620)	[36]
Punica granatum (pomegranate, crusts)	Room temperature Ultrasonication 24 h	20.12	Spherical and cubes	Antiproliferation effect and enhanced apoptosis against human breast adenocarcinoma cell line (MCF-7)	[37]
Honey	100 °C for 2 and 4 h	2.2	Nanowires	---	[38]
Streptomyces species (Gram-positive bacteria)	50 °C; 24 h	20–50	Spherical	Anticancer activity against human breast cancer cell lines (MCF-7)	[39]
Padina gymnospora (brown algae)	room temperature for 10 min	5–50	Truncated octahedral	Bactericidal activity against Escherichia coli, Lactococcus lactis, and Klebsiella pneumoniae	[40]
Sheep milk	room temperature for 3 h.	9	Spherical	---	[41]
Prunus x yedoensis (gum extract)	pH 8; gum extract concentrations of 7% and 8% 30 min	10–50	Spherical	Antifungal against Colletotrichum acutatum and Cladosporium fulvum	[42]

**Table 5:** In vitro antibacterial activity assays results of CH-AgNP samples. [Disk diffusion method (DDM); agar well diffusion method (AWD—mm); minimum inhibition concentration (MIC—μg/mL) and minimum bactericidal concentration (MBC—μg/mL)].

MICROORGANISMS		Honey Extract (100 mg/mL)				CH-AgNPs (30 °C)				CH-AgNPs (60 °C)				CH-AgNPs (90 °C)			
		DDM	AWD	MIC	MBC	DDM	AWD	MIC	MBC	DDM	AWD	MIC	MBC	DDM	AWD	MIC	MBC
**Gram positive**	*B. cereus* ATCC 14579	-	-	-	-	15	11	150	150	15	11	150	150	17	13	150	150
	*E. faecalis* ATCC 49452	-	-	-	-	14	11	150	150	16	12	150	150	16	12	150	150
	*S. aureus* ATCC 25923	-	-	-	-	16	13	150	150	16	13	75	150	18	14	75	150
	*S. mutans* ATCC 35668	-	-	-	-	18	14	75	75	19	15	75	75	20	16	75	75
	*S. salivarus* ATCC 13419	-	-	-	-	18	14	75	150	18	14	150	150	20	16	75	75
**Gram negative**	*A. baumannii* ATCC BA1609	-	-	-	-	14	11	150	150	15	12	150	150	17	13	150	150
	*E. coli* ATCC BAA 25–23	-	-	-	-	15	12	150	150	16	12	150	150	18	14	75	150
	*P. aeruginosa* ATCC 9070	-	-	-	-	14	11	150	150	15	11	150	150	15	12	150	150
	*S. Typhimurium* RSSK 95091	-	-	-	-	17	14	75	75	18	14	75	75	19	16	75	75
	*Y. enterocolitica* ATCC 27729	-	-	-	-	16	13	150	150	16	13	150	150	18	15	75	75

## Data Availability

Not applicable.

## Sample Availability

Samples of the compounds CH-AgNPs are available from the authors.
